# Early life characteristics and late life burden of cerebral small vessel disease in the Lothian Birth Cohort 1936

**DOI:** 10.18632/aging.101043

**Published:** 2016-09-19

**Authors:** Thalia S. Field, Fergus N. Doubal, Wendy Johnson, Ellen Backhouse, Caroline McHutchison, Simon Cox, Janie Corley, Alison Pattie, Alan J. Gow, Susan Shenkin, Vera Cvoro, Zoe Morris, Julie Staals, Mark Bastin, Ian J. Deary, Joanna M. Wardlaw

**Affiliations:** ^1^ University of British Columbia, S169-2211 Wesbrook Mall, Vancouver BC, V6T 2B5, Canada; ^2^ Division of Clinical Neurosciences, Centre for Clinical Brain Sciences, University of Edinburgh, Edinburgh, UK; ^3^ Centre for Cognitive Ageing and Cognitive Epidemiology, Department of Psychology, University of Edinburgh, Edinburgh, UK; ^4^ Centre for Clinical Brain Sciences, University of Edinburgh, Edinburgh, UK; ^5^ Department of Psychology, Heriot-Watt University, Edinburgh, UK; ^6^ Geriatric Medicine Unit, University of Edinburgh, Edinburgh, UK; ^7^ Department of Neuroradiology, NHS Lothian, Edinburgh, UK; ^8^ Department of Neurology and Cardiovascular Research Institute Maastricht (CARIM), Maastricht University Medical Center (MUMC), Maastricht, the Netherlands; ^9^ Brain Research imaging Centre, University of Edinburgh, Edinburgh, UK

**Keywords:** cerebral small vessel disease, stroke, MRI, risk factors, aging

## Abstract

It is unknown whether relations between early-life factors and overall health in later life apply to burden of cerebral small vessel disease (cSVD), a major cause of stroke and dementia. We explored relations between early-life factors and cSVD in the Lothian Birth Cohort, a healthy aging cohort. Participants were recruited at age 70 (N = 1091); most had completed a test of cognitive ability at age 11 as part of the Scottish Mental Survey of 1947. Of those, 700 participants had brain MRI that could be rated for cSVD conducted at age 73. Presence of lacunes, white matter hyperintensities, microbleeds, and perivascular spaces were summed in a score of 0-4 representing all MRI cSVD features. We tested associations with early-life factors using multivariate logistic regression. Greater SVD score was significantly associated with lower age-11 IQ (OR higher SVD score per SD age-11 IQ = .78, 95%CI 0.65-.95, p=.01). The associations between SVD score and own job class (OR higher job class, .64 95%CI .43-.95, p=.03), age-11 deprivation index (OR per point deprivation score, 1.08, 95%CI 1.00-1.17, p=.04), and education (OR some qualifying education, .60 95%CI .37-.98, p=.04) trended towards significance (p<.05 for all) but did not meet thresholds for multiple testing. No early-life factor was significantly associated with any one individual score component. Early-life factors may contribute to age-73 burden of cSVD. These relations, and the potential for early social interventions to improve brain health, deserve further study.

## INTRODUCTION

Cerebral small vessel disease (cSVD) is common, comprises 20% of ischemic stroke [1, 2] and contributes to 45% of dementias, but there is no effective treatment [3]. Neuroradiological features visible on structural magnetic resonance imaging (MRI) include white matter hyperintensities (WMH), lacunar infarcts, enlarged perivascular spaces, microbleeds and cerebral atrophy [3, 4]. Several demographic and clinical factors are associated with increased risk of cSVD, including hypertension [5] diabetes, hypercholesterolemia [6], obesity [7], smoking [8], dietary salt [9] and alcohol intake, but a large proportion of the variance in presence and severity of cSVD is unexplained [10]. Other potential risk factors for cSVD are not well characterized.

It is plausible that early-life factors may influence the development and impact of cSVD. Variables including high childhood IQ, educational attainment and higher childhood socioeconomic status have been associated with greater white matter structural integrity on MRI in older age [11]. These same early-life factors have also predicted lower incidence of conditions linked with cSVD in some studies, including cardiovascular disease, stroke, and dementia [12-15]. cSVD burden may therefore provide mechanistic links between early-life factors and late-life stroke and dementia incidence.

The SVD score is a 4-point scale developed to summarize total brain burden of cSVD. One point is given for the presence of each of moderate-severe WMH, one or more microbleeds, one or more lacunar infarcts, and moderate to severe burden of enlarged perivascular spaces [16]. The score shows specificity for small vessel infarcts over other stroke mechanisms [17], correlates with ambulatory blood pressure [16] and cognitive impairment [4, 18]. In the Lothian Birth Cohort 1936 (LBC1936), all four individual components of the SVD score have been found to load significantly on a “small vessel disease” latent variable.

Using the SVD score, we examined the relations between total brain burden of cSVD at age 73 years and early life factors in the LBC 1936, a longitudinal study of aging.

## RESULTS

Mean age of all participants at the time of MRI scan was 72.7 years (SD .7 years). Forty-seven percent were female. Mean age-11 IQ was 100.8 (SD 15.3), and 82% (81% male, 84% female) participants went on to obtain some form of qualifying education (O-level [completion at age 16], A-level [completion at age 18], semi-professional/ professional training or a higher degree).

Table 2 shows results of the multivariate logistic regressions. In all multivariate models, greater SVD score was significantly associated with lower age-11 IQ (Model 3, OR for higher SVD score per SD greater age-11 IQ = .78, 95% CI .65 - .95, p = .01). In the crude ordinal regression analysis (Supplementary Table e-2), each additional point on the SVD score was associated with a non-significant 6% decrease (95% CI -8 - 18%, p=.36) in standardized age-11 IQ. The associations between SVD score and own job class (Model 3, OR for higher versus lower class, .64, 95% CI .43 – .95, p=.03), age-11 deprivation index (Model 3, OR per point greater deprivation score, 1.08, 95% CI 1.00 - 1.17, p=.04), and level of education (Model 3 OR for O-level or some qualifying education versus not, .60 95% CI .37 - .98, p=.04) all showed trends towards significance (p<.05 for all) but did not meet the p=.01 threshold for multiple testing. None of the early-life factors was significantly associated with any of the individual SVD score components (Table 2).

**Table 1:** Characteristics of participants by SVD score

	SVD score				
	0	1	2	3	4
Overall, n(%)	302 (44.4)	249 (36.6)	98 (14.4)	27 (4.0)	4 (.6)
Age, mean, SD	72.50 (.75)	72.52 (.69)	72.63 (.61)	72.91 (.56)	72.79 (.68)
Female, n(%)	148 (49.0)	101 (40.6)	57 (58.2)	15 (55.6)	0 (0.0)
Age at leaving full-time education, y, mean (SD)	15.80 (1.14)	13.88 (1.14)	11.59 (1.00)	16.00 (1.39)	15.75 (1.50)
Number of years of full-time education, y, mean, SD	10.81 (1.15)	10.88 (1.14)	10.59 (1.00)	11.00 (1.39)	10.75 (1.50)
Education, highest qualification, n(%)					
No qualification	49 (16.2)	37 (15.0)	20 (20.4)	8 (29.6)	2 (50.0)
O-level/equivalent	115(38.1)	89 (36.0)	43 (43.9)	10 (37.0)	1 (25.0)
A-level/equivalent	56 (18.5)	41 (16.6)	9 (9.2)	4 (14.8)	1 (25.0)
Semiprofessional/professional	33 (10.9)	37 (15.0)	12 (12.2)	1 (3.7)	0 (0.0)
Degree	49 (16.2)	43 (17.4)	14 (14.3)	4 (14.8)	0 (0.0)
Education, dichotomized					
No qualification	49 (16.2)	37 (15.0)	20 (20.4)	8 (29.6)	2 (50.0)
O-level or higher	253 (83.8)	210 (85.0)	78 (79.6)	19 (70.4)	2 (50.0)
Own job class, n(%)					
I	64 (21.2)	51 (20.5)	11 (11.2)	11 (40.7)	0 (0)
II	121 (40.1)	99 (39.8)	37 (37.8)	4 (14.8)	1 (25.0)
IIIN	62 (20.5)	50 (20.1)	24 (24.5)	5 (18.5)	1 (25.0)
IIIM	37 (12.3)	42 (16.9)	24 (24.5)	6 (22.2)	2 (50.0)
IV	16 (5.3)	6 (2.4)	1 (1.0)	1 (3.7)	0 (0.0)
V	2 (.7)	1 (.4)	1 (1.0)	0 (0)	0 (0.0)
Dichotomized own job class, n(%)					
I-II	185 (61.3)	150 (60.2)	48 (49.0)	15 (55.6)	1 (25.0)
III-V	117 (38.7)	99 (39.8)	50 (51.0)	12 (44.4)	3 (75.0)
Age-11 IQ, mean (SD)	100.75 (14.28)	102.45 (14.27)	97.96 (18.21)	98.99 (14.11)	90.96 (8.46)
Age-11 deprivation index, mean (SD)	-.116 (2.39)	-.217 (2.07)	.453 (2.64)	-.228 (2.31)	−0.576 (1.39)
Father's job class, age-11, n(%)					
I	20 (6.6)	17 (6.8)	2 (2.0)	1 (3.7)	0 (0)
II	51 (16.9)	52 (20.9)	13 (13.3)	4 (14.8)	2 (50.0)
III	180 (59.6)	138 (55.4)	68 (69.4)	19 (70.4)	2 (50.0)
IV	32 (10.6)	23 (9.2)	11 (11.2)	3 (11.1)	0 (0)
V	19 (6.3)	19 (7.6))	4 (4.1)	0 (0)	0 (0)
Dichotomized father's job class, age-11, n(%)					
I-II	71 (23.5)	69 (27.7)	15 (15.3)	5 (18.5)	2 (50.0)
III-V	231 (76.5)	180 (72.3)	83 (84.7)	22 (81.5)	2 (50.0)

**Table 2:** Early life factor associations with dichotomized SVD score and components, multivariate logistic regression

Presence of moderate to severe cSVD						
	Model 1	p	Model 2	p	Model 3	p
Sex	.67 (.46 - .99)	.04	.68 (.46 - .98)	.05	.68 (.46 - 1.01)	.06
Age-11 IQ	.78 (.65-.94)	.01	.78 (.65 - .94)	.01	.78 (.65 - .95)	.01
Age-11 deprivation index	1.08 (1.00 – 1.17)	.04	1.08 (1.00 – 1.17)	.05	1.08 (1.00 – 1.17)	.04
Dichot. level of education, O-level or higher versus none	.58 (.36 - .94)	.03	.59 (.37 - .95)	.03	.60 (.37 - .98)	.04
Dichot. own job class I – II vs. III-V	.61 (.41 - .91)	.01	.61 (.41 - .91)	.01	.64 (.43 - .95)	.03
Dichot. father's job class, I – II vs. III-V	.62 (.38 – 1.02)	.06	.62 (.37-1.02)	.06	.63 (.38-1.04)	.07

Model 1: +Age and sex

Model 2: Model 1 + HTN

Model 3: Model 2 + Dyslipidemia + Smoking + Diabetes

**Table:** 

Presence of moderate to severe WMH						
	Model 1	p	Model 2	p	Model 3	p
Age-11 IQ	.84 (.70 – .99)	.04	.83 (.70 - .99)	.04	.84 (.70-1.00)	.05
Age-11 deprivation index	1.03 (0.95 – 1.11)	.49	1.03 (.95-1.11)	.48	1.02 (.95 – 1.11)	.56
Dichotomized level of education, O-level or higher versus none	.83 (.52 – 1.33)	.44	.86 (.42 - .88)	.52	.86 (.53 – 1.39)	.54
Dichotomized own job class I – II vs. III - V	.66 (.46-.95)	.02	.66 (.45 - .95)	.03	.71 (.49 – 1.04)	.08
Dichot father's job class, I – II vs. III - V	.71 (.45 – 1.11)	.14	.71 (.45 – 1.11_	.13	.73 (.46 – 1.16)	.18

**Table:** 

Presence of microbleeds						
	Model 1	p	Model 2	p	Model 3	p
Age-11 IQ	.93 (0.74 – 1.16)	.50	.93 (.74 – 1.16)	.50	.91 (.72 – 1.14)	.40
Age-11 deprivation index	1.03 (0.93 – 1.13)	.57	1.03 (.93-1.13)	.57	1.04 (.94 – 1.15)	.42
Dichotomized level of education, O-level or higher versus none	.55 (.32 - .95)	.03	.55 (.32 - .96)	.03	.57 (.32 - .99)	.05
Dichotomized own job class I – II vs. III - V	.72 (.45 – 1.15)	.17	.72 (.45 – 1.15)	.17	.70 (.43 – 1.13)	.70
Dichotomized father's job class, III - V vs. I - II	.80 (.45 – 1.43)	.46	.80 (.45 – 1.43)	.45	.79 (.44 – 1.42)	.43

**Table:** 

Presence of lacunes						
	Model 1	p	Model 2	p	Model 3	p
Age-11 IQ	.80 (.59 – 1.10)	.18	.80 (.58 – 1.10)	.17	.82 (.59 – 1.13)	.22
Age-11 deprivation index	.96 (.81 – 1.13)	.61	.96 (.81-1.13)	.62	.95 (.81 – 1.13)	.57
Dichotomized level of education, O-level or higher versus none	.78 (.33- 1.86)	.58	.81 (.34 – 1.94)	.64	.86 (.35 - 2.08)	.73
Dichotomized own job class I – II vs. III - V	.75 (.37 – 1.52)	.43	.76 (.37 – 1.53)	.44	.74 (.36 – 1.52)	.41
Dichotomized father's job class, I – II vs. III - V	1.67 (.79 – 3.52)	.18	1.66 (.79 – 3.52)	.19	1.71 (.80 – 3.65)	.17

**Table:** 

Presence of moderate to severe EPVS						
	Model 1	p	Model 2	p	Model 3	p
Age-11 IQ	1.04 (.89-1.21)	.63	1.04 (.89 – 1.21)	.63	1.04 (.89 – 1.21)	.63
Age-11 deprivation index	1.03 (.96 – 1.10)	.39	1.03 (.96-1.10)	.38	1.03 (.96 – 1.10)	.42
Dichotomized level of education, O-level or higher versus none	.83 (.55 – 1.25)	.37	.84 (.56 – 1.27)	.41	.84 (.56 – 1.27)	.41
Dichotomized own job class I – II vs. III - V	.95 (.69 – 1.29)	.72	.95 (.69 – 1.29)	.73	.98 (.71 – 1.36)	.92
Dichotomized father's job class, I – II vs. III - V	1.01 (.70 – 1.45)	.97	1.01 (.70 – 1.45)	.97	1.02 (.71-1.47)	.90

## DISCUSSION

In this large sample of generally healthy community-dwelling older members recruited from a single year-of-birth cohort, the total brain burden of cSVD at age 73 was associated with lower age-11 IQ, and showed theory-consistent trends towards association with age-11 deprivation index, own job class and highest qualifying education level. The SVD score was significantly associated with these early-life factors more consistently than any one individual MRI feature of cSVD. This is consistent with interpretation of SVD score as a latent construct. That is, it suggests that some underlying causal factor may influence development of each of these features of cSVD. If so, future research should seek to identify this causal factor.

Though conventional vascular risk factors, particularly hypertension and smoking, are associated with presence and burden of cSVD, we have previously found that these risk factors accounted for no more than 2% of variance of WMH, whereas their contributions to other forms of vascular disease, including coronary, carotid and peripheral large artery atheromatous disease, were much greater, explaining 70% of variance in the LBC1936 and related cohorts.[10] Our findings suggest that the effects of early-life deprivation and childhood IQ on overall health extends to burden of cSVD in later life, though their overall contributions to variance of cSVD is likely small (pseudo R^2^ = .03 and .01, respectively).

Relations between social characteristics and health are consistently observed, although there is less information on associations with cSVD than with other common disorders. The first reported association between socioeconomic status and overall health, in the landmark Whitehall study, was a strong association between employment class in the British Civil Service and age-adjusted mortality [32]. Evidence from subsequent cohorts has shown further associations between occupation class and cardiovascular disease and other chronic illnesses as well as age-adjusted mortality [33-36]. The roles of early-life socio-economic factors, including level of education, in establishing good health are also established [37]. Deprivation, both in utero [38] and in childhood [39, 40] is also associated with increased prevalence of cardiovascular and other chronic diseases and increased mortality.

The relations between social variables and brain health, however, are less well described. In the Aberdeen Birth Cohort 1936, WMH volumes were higher with “blue collar” than “white collar” paternal occupation grade [41]. Educational attainment is associated with lower prevalence of dementia [42], and has been shown in several cohorts to moderate the association between WMH and clinical expression of brain disease: patients with higher levels of education had better mean-level cognitive performance than less-educated patients with equally severe WMH on MRI [43, 44]. Educational attainment also had a dose-response association with cognitive impairment regardless of neuropathological features including neurofibrillary tangles, small vessel disease, and Lewy bodies [45]. However, few studies have explored or found direct associations between educational attainment and burden of cSVD [44] and we did not find a significant association that passed multiple testing thresholds between education and SVD score in this cohort.

Educational attainment, however, is closely linked with childhood IQ [46]. There is evidence that genetic and environmental influences on educational attainment can vary with IQ, with greater shared environmental influences on educational attainment at lower than higher IQs, but greater genetic influences on educational attainment at higher than lower IQs [47].

Having a higher IQ may be associated with interests and enjoyment of school that leads to seeking of further education, which in turn may lead to better employment opportunities and access to other social benefits associated with improved health. Family resources (which tend to vary with parental IQ) may be more likely to be funneled toward education when offspring show the academic potential that genetically-influenced IQ tends to reflect [37]. Childhood IQ has also been associated with burden of WMH and cortical thickness in older age [48, 49], and the association we have found between age-11 IQ and burden of SVD may also reflect better white matter structural integrity in those with higher age-11 IQ. This was previously observed in this cohort using fractional anisotropy derived from diffusion tensor MRI [11].

The precise mechanisms by which early socioeconomic factors and childhood intelligence may influence brain integrity in older age is unknown. While statistical adjustment for conventional vascular risk factors partially accounts for the effects of general health on brain health, there are other known and unknown variables that may further account for this association. Multiple studies have reported epigenetic associations for multiple socioeconomic factors, [50-52], poorer quality diet, aspects of environment that influence opportunities for and appeal of physical activity [53] and exposure to pollution and other toxins [54]. Socioeconomic status and childhood intelligence may also contribute to lifestyle choices impacting brain vascular health [55] – intelligence and greater educational attainment were associated with health-promoting behaviours [56] and earlier adoption of health-related recommendations [57] - and exposure or tolerance to allostatic load [58].

Though females had higher mean burden of cSVD than males, this finding is not consistent across cohorts [17, 59, 60]. In this study, the difference in burden of disease was not explained by younger school-leaving age in females, nor by differential mean burden of other early-life factors or vascular risk factors. (Supplementary Tables e-4 and e-5). There may be additional unidentified causes underlying this sex-dependent difference in cSVD burden in this sample, and sex difference may vary across samples. Alternatively, the findings could be statistical artifacts.

Strengths of our study were its sample size, availability of early-life data on older-age participants, absence of potential confounding effects of heterogeneous age of participants, and focus on one geographical region, which limited confounding effects due to local variations in socioeconomic strata. The latter two also limited potential to generalize beyond this group. Moreover, our analysis was limited by a concentration of lower SVD scores in our cohort, which likely reflected the good general health of this community-based, relatively young [30] and healthy aging cohort of voluntary participants; fuller associations between more severe cSVD and early-life factors may not have been completely characterized. The retrospective ascertainment of age-11 socioeconomic variables at age 70 may be subject to recall bias and error. We adjusted our significance threshold for multiple testing, though the possibility of Type I error remains. Our cohort's early childhood occurred during the Second World War, which could have impacted the development of cognitive ability or performance on cognitive ability tests as well as later-life health. However, the mean score on the Moray House Test No. 12 was higher in the population born in 1936 (Scottish Mental Survey 1947) than in the population born in 1921 (Scottish Mental Survey 1932) [61]. Further, relations between childhood IQ and adult intelligence have been reported in other cohorts and are likely generalizable. In another Lothian Birth Cohort born in 1921, intelligence at age 11 predicted intelligence at age 79, 87, and 90 [62, 63]; in the 1921 Aberdeen Birth Cohort, childhood intelligence also was associated with intelligence at age 78 – 81 [64], with correlations very consistent with those observed here. Heritability of intelligence in both the 1921 and 1936 cohorts is also similar at age 11, which would not be expected if there were adverse environmental influences on one of the cohorts [65].

## METHODS

### Participants

The LBC1936 has been previously described in detail [19]. Briefly, participants were born in 1936 and most completed the Moray House Test No. 12 intelligence test at age 11 as part of the 1947 Scottish Mental Survey, which tested 70805 out of a possible 75211 children from the total Scottish population born in 1936 [19]. Those living in the Lothian region of Scotland, mainly Edinburgh city, were recruited into a longitudinal study of aging at a mean age of 70 years, and 1091 consented to participate. From the original Wave 1 participants (mean age=69.5, SD = 0.8), 866 participants returned three years later for Wave 2 (mean age = 72.5, SD = 0.7), 700 of whom provided some usable brain MRI sequences and cognitive testing [20]. Twenty participants were removed for missing sequences. Primary reasons for attrition between Waves 1 and 2 were death, chronic incapacity, and permanent withdrawal [21]. Characteristics of Wave 2 participants with and without complete MRI data are summarized in Supplementary Table e-1. All participants provided written informed consent. The study was approved by Multi-Centre Research Ethics Committee for Scotland (MREC/01/0/56) and Lothian Research Ethics Committee (LREC/2003/2/29).

### Data

Data acquisition was described in detail previously [22]. Demographic details, sociodemographic background, educational history, and self-reported medical history were obtained by standardized interview between autumn 2007 and spring 2010.

Age-11 IQ scores were calculated from the Moray House Test No.12, which was administered on June 4, 1947, to almost all children attending school in Scotland who were born in 1936. Its correlation with the Stanford-Binet IQ score is ≈.80 [23]. Raw scores were adjusted for age in days at time of testing and placed on an IQ-type scale with a mean of 100 (SD=15) [22, 23]. Participants reported age at leaving school and educational attainment (none, O-level, complete at age 16; A-level, complete at age 18; semi-professional or professional, or degree), as well as their fathers’ numbers of years of full-time education. We calculated each person's number of years of full-time education.

Participants reported their own and their fathers’ principal occupations prior to retirement; married women also reported those of their spouses. Social class for fathers was ascertained using the General Register Office's Census, 1951 Classification of Occupations (coinciding approximately with the middles of the fathers’ career). Social class was ascertained for participants using the Office of Population Censuses and Surveys’ Classification of Occupations, 1980 (coinciding approximately with participants’ mid-career). Both are scales from I-V, from professional to unskilled labor. For married women, the husband's social class was used if it was higher. Participants reported information on their childhood homes, including the numbers of people living in their home, numbers of rooms and toilets, numbers of people sharing toilets and whether the toilets were indoor or outdoor. Number of people per room was calculated and standardized; the other two variables were standardized separately, and a composite measure of age-11 deprivation was formed by summing these standardized variables [22].

### Neuroimaging and interpretation

Brain imaging acquisition for LBC1936 has been described previously in detail [24]. All subjects were scanned on the same 1.5T GE Signa HDx scanner operating in research mode. The SVD score components were ascertained from axial T2, T2*, fluid-attenuated inversion recovery (FLAIR) and T1-weighted sequences.

Images were rated by a certified and registered neuroradiologist (ZM) blinded to clinical information. Presence of lacunes, WMH, microbleeds and perivascular spaces were rated according to established protocol, published previously, using validated visual scales [24-26]. A second experienced certified and registered neuroradiologist (JMW) re-rated 20% of scans selected at random and consensus was made on disagreements. Intraclass correlation coefficient for WMH was 0.96 [27], and intra- and inter-rater kappa statistics for perivascular spaces were 0.8-0.9 [27]. Inter-rater kappa for microbleeds in a separate cohort was 0.68 [25]. The lower inter-rater agreement for microbleeds likely reflects the greater location choices of the BOMBS rating scale, not the presence versus absence of microbleeds for which the observer agreement was high.

Rating scales for the variables above were converted to dichotomous point scores and summed to create the SVD score [4, 16-18].

### Statistical analysis

First, descriptive characteristics were calculated using means and standard deviations (SD) or counts and percentages as appropriate (Table 1). As there were small amounts of missing data for many of the variables that would have reduced the sample size with listwise deletion, their status was tested using Little's MCAR test to assess for completely random missingness. We used expectation maximization estimates to impute missing values [29]. Neither own job class nor fathers’ job class met criteria for complete missingness. We considered it likely they met criteria for missingness at random, however, and imputed them as well, rounding to the nearest integer or, in the case of own job class, half-integer as appropriate. Five missing values were imputed for own social class and 20 for fathers’ job class, all of which had a central tendency. To compare associations with more- and less-deprived states, we also examined dichotomized scores for participant's job class, father's job class (both into unskilled [IV, V] and skilled [I, II, IIIN, IIIM]) and highest educational attainment (did not finish primary education and O-level or higher). With the exception of own job class, which was negatively skewed with only 4.6% of participants with classes of IV or V, these variables were approximately normally distributed.

**Figure 1:**
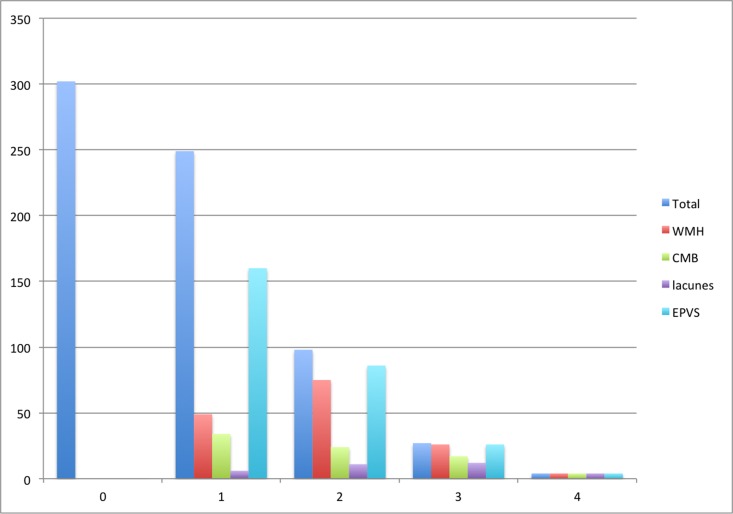
Number of participants with each SVD score and score component

Few in the cohort had high SVD scores (0.6% had an SVD score of 4, 4% a score of 3, 14% a score of 2, 37% a score of 1 and 44% a score of 0; Figure 1), which likely reflected the generally good health and relatively young age of this community-dwelling healthy aging cohort [22]. Given the skewed distribution of SVD scores in the cohort, we explored the relations between early life factors and the SVD scores using logistic regression with a dichotomized SVD score: SVD 0-1 (“no or mild disease”) and SVD 2-4 (“moderate-severe disease”).

We performed univariate logistic regression for differences in early-life factors for higher and lower SVD scores and for presence of each individual component; we then included factors with p < .10 in multivariate logistic regression. We used three multivariate models, first adjusting age and sex, then adding hypertension, then other vascular risk factors (history of dyslipidemia, diabetes and smoking status). The covariates in the models were chosen as all are known to account at least to some degree independently for variance in burden of cSVD on MRI, both in this cohort and in others [10, 17, 30, 31]. To offset effects of multiple testing, we considered effects to be significant at p values of .01. Analyses were performed using SPSS 23.0 (Armonk, NY).

## CONCLUSIONS

We observed that age-11 IQ was associated with greater burden of cSVD on MRI in later life, and that age-11 deprivation index and low social class showed similar trends. This might help explain the known association between such factors and risk of stroke and dementia. The relations between early life factors were more strongly associated with total brain burden of SVD than any one neuroradiological component of WMH, cerebral microbleeds, lacunes or enlarged perivascular spaces.

Our findings suggest that childhood intelligence may be a “risk factor” for cSVD in later life. This observation, as well as the relations between other early-life factors and burden of cSVD, deserves further study.

## SUPPLEMENTARY MATERIAL TABLES


