# Supplementary material for: Early life characteristics and late life burden of cerebral small vessel disease in the Lothian Birth Cohort 1936
Source: Aging (Albany NY). 2016 Sep 19;8(9):2039–51. doi: 10.18632/aging.101043 (PMC5076451; doi:10.18632/aging.101043)
Supplement: Supplementary file 1 [file aging-08-2039-s001.pdf]

SUPPLEMENTARY MATERIAL

Supplementary Table e-1. Characteristics of all Wave 2 participants and of those with complete MRI data

	Missing MRI data (n=186)	With complete MRI data (n=680)
Age, mean, SD	72.53 (.73)	72.55 (.71)
Female, n(%)	97 (52.2)	321 (47.2)
Age at leaving full-time education, y, mean (SD)	15.69 (1.14)	15.81 (1.14)
Number of years of full-time education, y, mean, SD	10.69 (1.15)	10.81 (1.14)
Education, highest qualification, n(%)	*Missing=0*	*Missing=2*
No qualification	37 (19.9)	116 (17.1)
O-level/equivalent	72 (38.7)	258 (38.1)
A-level/equivalent	33 (17.7)	111 (16.4)
Semiprofessional/professional	21 (11.3)	83 (12.2)
Degree	23 (12.4)	110 (16.2)
Own job class, n(%)	*Missing=5*	*Missing=11*
I	29 (15.6)	137 (20.5)
II	71 (38.2)	252 (37.7)
IIIN	44 (23.7)	141 (21.1)
IIIM	32 (17.2)	111 (16.6)
IV	4 (2.2)	24 (3.6)
V	1 (.5)	4 (.6)
Age-11 IQ, mean (SD)	*Missing=0*<br>100.05 (14.78)	*Missing=36*<br>100.84 (15.32)
Age-11 deprivation index	*Missing=0*<br>-.117 (2.297)	*Missing=7*<br>-.075 (2.328)
Father's job class, age-11, n(%)	*Missing=20*	*Missing=58*
I	16 (8.6)	40 (6.4)
II	31 (16.7)	122 (19.6)
III	88 (47.3)	351 (56.4)
IV	12 (6.5)	67 (10.8)
V	19 (10.2)	42 (6.8)
Father's number of yrs of education, age-11, mean (SD)	*Missing=45*<br>9.84 (2.48)	*Missing=147*<br>10.02 (2.21)

Supplementary Table e-2. Early life factor associations with total SVD score in ordinal regression analyses

	**Crude OR (95% CI)**	**p**
Female	.97 (.74-1.29)	.86
Age	1.25 (1.03 – 1.53)	.03
Highest educational qualification		
Degree	.97 (.74 – 1.29)	.26
Semi-professional/professional	1.23 (.77 – 1.96)	.69
A-level/equivalent	.94 (.81 – 1.08)	.37
O-level/equivalent	1.11 (.65 – 1.89)	.71
None	Ref	
Education dichotomized, O-level or above vs. none	.76 (.53 – 1.11)	.16
Own job class		
5	1.01 (.16 – 6.55)	.99
4	.44 (.18 – 1.07)	.07
3.5	1.84 (1.15 – 2.92)	.01
3	1.17 (.75 – 1.81)	.48
2	.98 (.66 – 1.45)	.92
1	Ref	
Own job class dichotomized III - V vs. I - II	.76 (.57-1.01)	.54
Age-11 deprivation score per point	1.03 (.81 – 1.10)	.30
Age-11 IQ, per SD	.94 (.82-1.08)	.36
Father's job class		
5	1.17 (.51 – 2.67)	.71
4	1.40 (.66 – 2.91)	.39
3	1.52 (.82 – 2.85)	.18
2	1.46 (.74 – 2.89)	.28
1	Ref	
Father's job class dichotomized III - V vs. I - II	.90 (.65-1.26)	.54
Years of education	.97 (.86 – 1.10)	.66
Age left education	.97 (.86-1.10)	.67

Supplementary Table e-3. **Early life factor associations with dichotomized SVD score and components, univariate logistic regression analyses**

**Presence of Moderate to Severe cSVD**		
	**Crude OR (95% CI)**	**p**
Female	1.53 (1.04 – 2.25)	.03
Age	1.43 (1.09 – 1.89)	.01
Education, highest qualification, n(%)<br>No qualification<br>O-level/equivalent<br>A-level/equivalent<br>Semiprofessional/profess.<br>Degree	 <br>Ref<br>.76 (.46 – 1.27)<br>.41 (.21 - .83)<br>.53 (.26 - 1.10)<br>.56 (.29 – 1.08)	 <br><br>.29<br>.01<br>.09<br>.08
Education, dichotomized<br>No qualification vs. O-level or higher	.61 (.38 - .98)	.04
Own job class<br>5<br>4<br>3.5<br>3<br>2<br>1	 1.74 (.17 – 17.53)<br>.48 (.10 – 2.17)<br>2.12 (1.15 – 3.91)<br>.28 (.76 – 2.57)<br>1.00 (.57 – 1.75)	 .64<br>.34<br>.02<br>.28<br>.99
Dichotomized own job class<br>.I – II vs. III - V	.64 (.43 - .93)	.02
Age-11 deprivation score	1.08 (1.00 – 1.16)	.06
Age-11 IQ, per SD	0.80 (0.66 – 0.96)	.02
Father's job class,<br>5<br>4<br>3<br>2<br>1	 1.30 (.27 - 6.20)<br>3.14 (.84 – 11.69)<br>3.45 (1.04 – 11.46)<br>2.28 (.64 – 8.14)<br>Ref	 .74<br>.09<br>.04<br>.21
Dichotomized father's job class<br>I – II vs. III - V	.60 (.37 -.99)	.05
Years of education	0.88 (0.74– 1.05)	.16
Age left education	0.88 (0.74 – 1.05)	.17

Supplementary Table e-3. Continue

Presence of of moderate to severe WMH		
	**Crude OR (95% CI)**	**p**
Female	1.57 (1.09 – 2.25)	.02
Age	1.33 (1.03 – 1.72)	.03
Education, highest qualification, n(%)		
No qualification	Ref	
O-level/equivalent	1.01 (.61 – 1.68)	.96
A-level/equivalent	52 (.27 – 1.03)	.06
Semiprofessional/profess	.99 (.51 – 1.90)	.96
Degree	.87 (.47 – 1.60)	.65
Education, dichotomized<br>No qualification vs. O-level or higher	.88 (.55 – 1.39)	.57
Dichotomized own job class<br><br>I – II vs. III - V	.68 (.48 - .98)	.04
Age-11 deprivation score	1.02 (.95-1.10)	.57
Age-11 IQ, per SD	.85 (0.71 – 1.01)	.07
Dichotomized father's job class<br>I – II vs. III - V	.69 (.44-1.09)	.11
Years of education	.97 (.83-1.14)	.70
Age left education	.97 (0.82 – 1.13)	.67
**Presence of microbleeds**		
Female	.83 (.52-1.33)	.43
Age	1.55 (1.1I - II.18)	.01
Education, highest qualification, n(%)		
No qualification	Ref	.09
O-level/equivalent	.60 (.32 – 1.09)	.12
A-level/equivalent	.55 (.26 – 1.18)	.10
Semiprofessional/profess	.48 (.20 – 1.15)	.02
Degree	.36 (.15 - .84)	
Education, dichotomized<br>No qualification vs. O-level or higher	.52 (.30 - .90)	.02
Dichotomized own job class<br><br>I – II vs. III - V	.69 (.43 – 1.11)	.12
Age-11 deprivation score	1.54 (.69-3.45)	.29
Age-11 IQ, per SD	.90 (.72-1.13)	.36
Dichotomized father's job class<br>I – II vs. III - V	.79 (.44 - 1.41)	.43
Years of education	.91 (.74-1.13)	.40
Age left education	.92 (.74-1.13)	.41

Supplementary Table e-3. Continue

**Presence of lacunes**		
	**Crude OR (95% CI)**	**p**
Female	.93 (.46-1.88)	.84
Age	1.45 (.88-2.41)	.15
Education, highest qualification, n(%)		
No qualification	Ref	
O-level/equivalent	.89 (.35 – 2.28)	.81
A-level/equivalent	.74 (.23 – 2.39)	.61
Semiprofessional/profess	.19 (.02 – 1.57)	.12
Degree	.90 (.29 – 2.76)	.85
Education, dichotomized No qualification vs. O-level or higher	.76 (.32 – 1.78)	.52
Dichotomized own job class I – II vs. III - V	.74 (.37 – 1.49)	.39
Age-11 deprivation score	.83 (.27-2.56)	.75
Age-11 IQ, per SD	.79 (.58-1.09)	.15
Dichotomized father's job class I – II vs. III - V	.1.64 (.78 – 3.46)	.19
Years of education	1.08 (.80-1.46)	.61
Age left education	1.08 (0.803-1.462)	.60
**Presence of moderate to severe EPVS**		
Female	.88 (0.65-1.19)	.41
Age	.98 (.90-1.06)	.54
Education, highest qualification, n(%)		
No qualification	Ref	
O-level/equivalent	.79 (.51 – 1.23)	.30
A-level/equivalent	.75 (.44 – 1.27)	.29
Semiprofessional/profess	.90 (.51 – 1.59)	.71
Degree	.85 (.50 – 1.44)	.55
Education, dichotomized No qualification vs. O-level or higher	.81 (.54 – 1.21)	.30
Dichotomized own job class I – II vs. III - V	.93 (.68 – 1.27)	.64
Age-11 deprivation score	1.23 (.74-2.06)	.42
Age-11 IQ, per SD	1.03 (.88-1.20)	.75
Dichotomized father's job class I – II vs. III - V	1.01 (.70 – 1.44)	.96
Years of education	.99 (.87-1.14)	.91
Age left education	1.00 (.87-1.14)	.96

Supplementary Table e-4. **Baseline characteristics by sex**

	Male	Female	p
Age-11 IQ, mean (SD)	99.08 (15.51)	100.99 (13.53)	.008
Age-11 Deprivation Index, mean (SD)	.130 (2.544)	-.137 (2.239)	.02
Number of years of full-time education, mean (SD)	10.75 (1.16)	10.73 (1.10)	.18
Highest educational attainment, n(%)			
None	105 (19.2)	88 (16.2)	<.0001
O-level	177 (32.4)	253 (46.7)	
A-level	111 (20.3)	71 (13.1)	
Semiprof/prof	60 (11.0)	66 (12.2)	
Degree	94 (17.2)	64 (11.8)	
Own job class, n (%)			
			<.0001
I	95 (17.2)	95 (17.5)	
II	196 (35.8)	225 (41.4)	
IIIN	66 (12.0)	182 (33.5)	
IIIM	152 (27.7)	36 (6.6)	
IV	34 (6.2)	4 (.7)	
V	5 (0.9)	1 (.2)	
Dichot. own job, n(%)			
I-II	291 (53.1)	320 (58.9)	.06
III-V	257 (46.9)	223 (41.1)	
Father's job class, n (%)			
I			
II	33 (6.0)	35 (6.4)	.05
III	103 (18.8)	89 (16.4)	
IV	340 (62.0)	323 (59.5)	
V	33 (6.0)	60 (11.0)	
	39 (7.1)	36 (6.6)	
Dichot. father's job class, n (%)			.48
I-II	136 (24.8)	124 (22.8)	
III-V	412 (75.2)	419 (77.2)	
Mean age at MRI, y (SD)	72.71 (.71)	72.76 (.74)	.55
Hypertension, n (%)	218 (39.8)	215 (39.6)	.95
Diabetes, n (%)	63 (14.1)	32 (7.7)	.003
Dyslipidemia, n (%)	203 (37.1)	183 (33.7)	.25
Smoking, n (%)			
Never	220 (40.1)	281 (51.7)	<.0001
Former	268 (48.9)	197 (36.3)	
Current	60 (10.9)	65 (12.0)	

Supplementary Table e-5. Multivariable logistic regression, association between sex and burden of cSVD

a. Univariate

Moderate to severe cSVD[a]		Sig.	Exp(B)	95% Confidence Interval for Exp(B)	
				Lower Bound	Upper Bound
1.00	Intercept	.000			
	Male sex	.030	.653	.444	.960
	Female sex				

b. + Highest qualifying education

Moderate to severe cSVD[a]		p	Exp(B)	95% Confidence Interval for Exp(B)	
				Lower Bound	Upper Bound
1.00	Intercept	.000			
	Male sex	.023	.632	.425	.938
	Female sex				
	No qualifying ed	.069	1.839	.953	3.550
	0-level	.444	1.261	.697	2.280
	A-level	.393	.719	.337	1.533
	Semi-pro/pro	.799	.903	.413	1.975
	Degree				

c. +Own job class ("Her Majesty's Stationery Office [HMSO] social class")

SVD dichot[a]		Sig.	Exp(B)	95% Confldence Interval for Exp(B)	
				Lower Bound	Upper Bound
1.00	Intercept	.496			
	Male sex	.005	.529	.340	.824
	Female sex				
	No qualifying ed	.463	1.328	.622	2.834
	0-level	.954	.980	.500	1.923
	A-level	.320	.667	.301	1.481
	Semi-pro/pro	.815	.909	.408	2.024
	Degree				
	HMSO I	.681	.609	.057	6.478
	HMSO II	.688	.622	.061	6.338
	HMSO lII N	.809	.751	.073	7.687
	HMSO lIlM	.734	1.494	.148	15.076
	HMSO IV	.468	.368	.025	5.474
	HMSO V				

Supplementary Table e-5. Continue

d. +Smoking

Moderate to severe cSVD[a]		p	Exp(B)	95% Confidence Interval for Exp(B)	
				Lower Bound	Upper Bound
1.00	Intercept	.646			
	Male sex	.007	.540	.346	.843
	Female sex				
	No qualifying ed	.424	1.364	.637	2.917
	O.level	.972	1.012	.513	1.998
	A-level	.393	.704	.315	1.574
	Semi-pro/pro	.824	.913	.410	2.034
	Degree				
	HMSO I	.739	.667	.061	7.273
	HMSO II	.747	.680	.065	7.110
	HMSO IIIN	.816	.757	.072	7.923
	HMSO IIIM	.701	1.581	.153	16.356
	HMSO IV	.485	.379	.025	5.765
	HMSO V				
	Never smoked	.369	.734	.373	1.442
	Former smoker	.169	.613	.306	1.230
	Current smoker				

e. + Hypertension

Moderate to severe cSVD[a]		p	Exp(B)	95% Confidence Interval for Exp(B)	
				Lower Bound	Upper Bound
1.00	Intercept	.819			
	Male sex	.007	.541	.346	.845
	emale sex				
	No qualifying ed	.486	1.312	.611	2.817
	O.level	.995	.998	.505	1.974
	A-level	.381	.697	.311	1.561
	Semi·pro/pro	.788	.896	.401	2.002
	Degree				
	HMSO I	.701	.626	.057	6.818
	HMSO II	.708	.638	.061	6.667
	HMSO IIIN	.777	.713	.068	7.446
	HMSO IIIM	.743	1.477	.143	15.252
	HMSO IV	.467	.364	.024	5.534
	HMSO V				
	Never smoked	.341	.720	.366	1.416
	Former smoker	.157	.604	.301	1.214
	Current smoker				
	No hypertension	.112	.724	.486	1.078
	Hvpertenslon				

Supplementary Table e-5. Continue

f. + Father's job class

Moderate to severe cSVD[a]		p	Exp(B)	95% Confidence Interval for Exp(B)	
				Lower Bound	Upper Bound
1.00	Intercept	.437			
	age 11 deprivation score	.286	1.047	.962	1.138
	Male sex	.006	.531	.338	.834
	Female sex				
	No degree	.683	1.177	.538	2.575
	O-level	.688	.865	.427	1.753
	A-level	.305	.651	.286	1.478
	Semi-pro/pro	.632	.820	.364	1.846
	Degree				
	HSMO I	.696	.616	.054	7.011
	HMSO ll	.669	.594	.055	6.442
	HMSO lllN	.711	.637	.059	6.922
	HMSO lllM	.815	1.326	.125	14.081
	HMSO IV	.425	.326	.021	5.104
	HMSO V				
	Never smoked	.391	.742	.375	1.467
	Former smoker	.229	.649	.321	1.312
	Current smoker				
	No hypertension	.077	.695	.465	1.040
	Hypertension				
	Father's job class I	.989	1.012	.201	5.087
	Father's job II	.174	2.276	.695	7.458
	Father's job Ill	.048	3.001	1.010	8.918
	Father's job IV	.166	2.371	.698	8.050
	Father's job V				

SUPPLEMENTARY REFERENCES

1. Satizabal CL, Beiser AS, Chouraki V, Chene G, Dufouil C, Seshadri S. Incidence of Dementia over Three Decades in the Framingham Heart Study. N Engl J Med 2016;374:523-532.
2. Dufouil C, Alperovitch A, Tzourio C. Influence of education on the relationship between white matter lesions and cognition. Neurology 2003;60:831-836.
3. Lane EM, Paul RH, Moser DJ, Fletcher TD, Cohen RA. Influence of education on subcortical hyperintensities and global cognitive status in vascular dementia. J Int Neuropsychol Soc 2011;17:531-536.
4. Farfel JM, Nitrini R, Suemoto CK, et al. Very low levels of education and cognitive reserve: a clinicopathologic study. Neurology 2013;81:650-657.
5. Deary IJ, Strand S, Smith P, Fernandes C. Intelligence and educational achievement. Intelligence 2007;35:13-21.
6. Johnson W, Deary IJ, Iacono WG. Genetic and Environmental Transactions Underlying

Educational Attainment. Intelligence 2009;37:466-478.
7. Valdes Hernandez Mdel C, Booth T, Murray C, et al. Brain white matter damage in aging and cognitive ability in youth and older age. Neurobiol Aging 2013;34:2740-2747.
8. Karama S, Bastin ME, Murray C, et al. Childhood cognitive ability accounts for associations between cognitive ability and brain cortical thickness in old age. Mol Psychiatry 2014;19:555-559.
9. Mathers JC, Strathdee G, Relton CL. Induction of epigenetic alterations by dietary and other environmental factors. Adv Genet 2010;71:3-39.
10. McGuinness D, McGlynn LM, Johnson PC, et al. Socio-economic status is associated with epigenetic differences in the pSoBid cohort. Int J Epidemiol 2012;41:151-160.
11. Borghol N, Suderman M, McArdle W, et al. Associations with early-life socio-economic position in adult DNA methylation. Int J Epidemiol 2012;41:62-74.
12. Giles-Corti B, Donovan RJ. The relative influence of individual, social and physical environment determinants of physical activity. Soc Sci Med 2002;54:1793-1812.
13. Prochaska JD, Nolen AB, Kelley H, Sexton K, Linder SH, Sullivan J. Social Determinants of Health in Environmental Justice Communities: Examining Cumulative Risk in Terms of Environmental Exposures and Social Determinants of Health. Hum Ecol Risk Assess 2014;20:980-994.
14. Deary IJ, Weiss A, Batty GD. Intelligence and Personality as Predictors of Illness and Death: How Researchers in Differential Psychology and Chronic Disease Epidemiology Are Collaborating to Understand and Address Health Inequalities. Psychol Sci Public Interest 2010;11:53-79.
15. Barbeau EM, Krieger N, Soobader MJ. Working class matters: socioeconomic disadvantage, race/ethnicity, gender, and smoking in NHIS 2000. Am J Public Health 2004;94:269-278.
16. Cutler DM, Lleras-Muney A. Education and Health: Evaluating Theories and Evidence. National Bureau of Economic Research Working Paper Series 2006;No. 12352.
17. van Dijk EJ, Prins ND, Vrooman HA, Hofman A, Koudstaal PJ, Breteler MM. Progression of cerebral small vessel disease in relation to risk factors and cognitive consequences: Rotterdam Scan study. Stroke; a journal of cerebral circulation 2008;39:2712-2719.
18. Benavente O, Pearce LA, Andersen D, Bazan C, Hart RG. MRI Predictors of Stroke Recurrence in Patients With Recent Lacunar Stroke: The SPS3 Trial Stroke; a journal of cerebral circulation 2014;45:A66.
